# 5-Hy­droxy-8,8-dimethyl-10-(2-methyl­but-3-en-2-yl)-2*H*,6*H*-7,8-dihydro­pyrano[3,2-*g*]chromene-2,6-dione

**DOI:** 10.1107/S1600536811001565

**Published:** 2011-01-15

**Authors:** Hoong-Kun Fun, Tawanun Sripisut, Surat Laphookhieo, Suchada Chantrapromma

**Affiliations:** a X-ray Crystallography Unit, School of Physics, Universiti Sains Malaysia, 11800 USM, Penang, Malaysia; b Natural Products Research Laboratory, School of Science, Mae Fah Luang University, Tasud, Muang Chiang Rai 57100, Thailand; c Crystal Materials Research Unit, Department of Chemistry, Faculty of Science, Prince of Songkla University, Hat-Yai, Songkhla 90112, Thailand

## Abstract

In the title compound, C_19_H_20_O_5_, the pyran ring is in an envelope conformation, whereas the benzene and dihydro­pyran ring system is planar with an r.m.s. deviation of 0.0190 (1) Å. The hy­droxy group is coplanar with the attached benzene ring [r.m.s. deviation = 0.0106 (1) Å]. An intra­molecular O—H⋯O hydrogen bond generates an *S*(6) ring motif. In the crystal, mol­ecules are linked into chains along the *b* axis by weak C—H⋯O inter­actions. These chains are stacked along the *a* axis. C—H⋯π and weak π–π inter­actions [centroid–centroid distance = 3.7698 (7) Å] are also observed.

## Related literature

For bond-length data, see: Allen *et al.* (1987 ▶). For hydrogen-bond motifs, see: Bernstein *et al.* (1995 ▶) and for ring conformations, see: Cremer & Pople (1975 ▶). For background to Rutaceae plants, coumarins and their biological activity, see: Kongkathip *et al.* (2005 ▶); Laphookhieo *et al.* (2009 ▶); Maneerat *et al.* (2010 ▶); Huang *et al.* (1997 ▶); Su *et al.* (2009 ▶); Tangyuenyongwatthana *et al.* (1992 ▶); Yenjai *et al.* (2000 ▶).
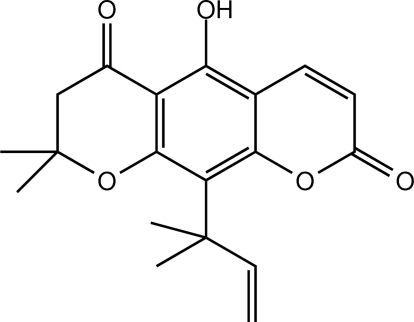


## Experimental

### 

#### Crystal data

C_19_H_20_O_5_
*M* *_r_* = 328.35
Monoclinic, 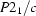

*a* = 10.2239 (2) Å
*b* = 11.3090 (3) Å
*c* = 13.8764 (3) Å
β = 93.108 (1)°
*V* = 1602.06 (6) Å^3^
*Z* = 4
Cu *K*α radiation
μ = 0.81 mm^−1^
*T* = 100 K
0.43 × 0.43 × 0.33 mm

#### Data collection

Bruker APEX DUO CCD area-detector diffractometer
Absorption correction: multi-scan (*SADABS*; Bruker, 2009 ▶) *T*_min_ = 0.721, *T*_max_ = 0.774
48432 measured reflections
3114 independent reflections
3088 reflections with *I* > 2σ(*I*)
*R*_int_ = 0.027

#### Refinement

*R*[*F* ^2^ > 2σ(*F* ^2^)] = 0.047
*wR*(*F* ^2^) = 0.147
*S* = 1.29
3114 reflections
234 parameters
H atoms treated by a mixture of independent and constrained refinement
Δρ_max_ = 0.71 e Å^−3^
Δρ_min_ = −0.84 e Å^−3^

### 

Data collection: *APEX2* (Bruker, 2009 ▶); cell refinement: *SAINT* (Bruker, 2009 ▶); data reduction: *SAINT*; program(s) used to solve structure: *SHELXTL* (Sheldrick, 2008 ▶); program(s) used to refine structure: *SHELXTL*; molecular graphics: *SHELXTL*; software used to prepare material for publication: *SHELXTL* and *PLATON* (Spek, 2009 ▶).

## Supplementary Material

Crystal structure: contains datablocks global, I. DOI: 10.1107/S1600536811001565/bq2271sup1.cif

Structure factors: contains datablocks I. DOI: 10.1107/S1600536811001565/bq2271Isup2.hkl

Additional supplementary materials:  crystallographic information; 3D view; checkCIF report

## Figures and Tables

**Table 1:** Hydrogen-bond geometry (Å, °) *Cg*1 is the centroid of the C1–C5/O1ring.

*D*—H⋯*A*	*D*—H	H⋯*A*	*D*⋯*A*	*D*—H⋯*A*
O5—H1*O*5⋯O4	0.93 (2)	1.66 (2)	2.5361 (14)	155 (2)
C9—H9*B*⋯O3^i^	0.97	2.36	3.2621 (17)	155
C16—H16*B*⋯O5^ii^	0.96	2.59	3.4982 (17)	159
C16—H16*C*⋯O2	0.96	2.34	2.9441 (16)	121
C15—H15*B*⋯*Cg*1^iii^	0.97 (2)	2.83 (2)	3.5908 (16)	136.7 (15)

Symmetry codes: (i) 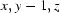; (ii) 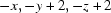; (iii) 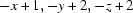.

**Fig. 1.**
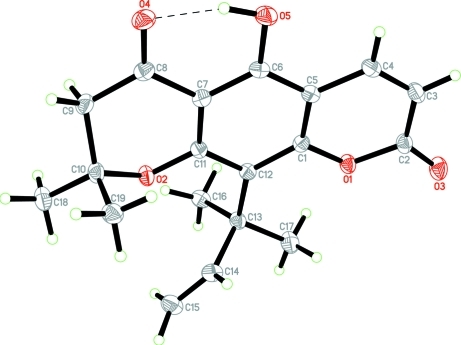
The structure of (I), showing 50% probability displacement ellipsoids and the atom-numbering scheme. O—H···O hydrogen bond is shown as dashed line.

**Fig. 2.**
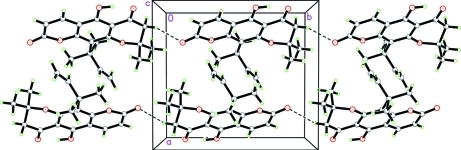
The crystal packing of (I) viewed along the c axis, showing chains along the b axis. Hydrogen bonds are shown as dashed lines.

## supplementary crystallographic information

### Comment

Rutaceae plants are the rich sources of coumarins and carbazole alkaloids. Many of them have been isolated from several genera of Rutaceae especially from *Clausena* genus (Laphookhieo *et al.*, 2009; Maneerat *et al.*, 2010; Tangyuenyongwatthana *et al.*, 1992) and some of these compounds show interesting pharmacological activities (Yenjai *et al.*, 2000). During our on-going research on bioactive natural products from Thai medicinal plants, the title pyranocoumarin which known as clausenidin (Huang *et al.*, 1997) was isolated from the roots of *C. excavata* which were collected from Suratthani province in the southern part of Thailand. Previous reports have found that clausenidin displayed anti-HIV-1 activity in a syncytial assay (Kongkathip *et al.*, 2005) and cytotoxicity against four human cancer cell lines (A549, MCF7, KB and KB-VIN) (Su *et al.*, 2009). We report herein the crystal structure of the title pyranocoumarin (I).

Fig. 1 shows that in the structure of (I), the pyran ring (C7–C11/O2) adopts an envelope conformation with the puckering atom C10 having deviation of 0.3279 (15) Å, and puckering parameters Q = 0.4648 (14) Å, θ = 123.32 (17)° and φ = 204.32° (Cremer & Pople, 1975). The benzene and dihydro-pyran ring system (C1–C7/C11-C12/O1) is planar with the *r.m.s*. 0.0190 (1) Å. The hydroxy group are planarly attached to the benzene ring. The orientation of the 2-methyl-but-3-enyl [C13–C17] side chain with respect to the benzene ring is indicated by the torsion angle of C12–C13–C14–C15 = 138.93 (16)°, indicating a (+)-anticlinal conformation (Fig. 1). Intramolecular O5—H1O5···O4 hydrogen bond (Table 1) generates an S(6) ring motif (Fig. 1 and Table 1) (Bernstein *et al.*, 1995). The bond distances in (I) are within normal ranges (Allen *et al.*, 1987).

The crystal packing of (I) is stabilized by intermolecular C—H···O and C—H···π weak interactions (Table 1). The molecules are linked into chains along the *b* axis and these chains are stacked along the *a* axis (Fig. 2 and Table 1). π–π interactions with the Cg_1_···Cg_2_ distance = 3.7698 (7) Å (symmetry code: -x, 2-y, 2-z) are observed; Cg_1_ and Cg_2_ are the centroids of C1–C5/O1 and C1/C5–C7/C11-C12 rings, respectively.

### Experimental

The roots of *C. excavata* (3.98 Kg) were successively extracted with CH_2_Cl_2_ over the period of 3 days at room temperature to provide the crude CH_2_Cl_2_ extract which was subjected to quick column chromatography (QCC) over silica gel eluted with a gradient of hexane-EtOAc (100% hexane to 100% EtOAc) to provide twenty-one fractions (A-U). Fraction G (10.68 g) was further separated by QCC with a gradient of 10% EtOAc-hexane to 100% EtOAc to give seven subfractions (G1-G7). Subfraction G4 (1.82 g) was subjected to repeated column chromatography using 6% EtOAc-hexane to yield the yellow solid of the title compound (30.0 mg). Yellow block-shaped single crystals of the title compound suitable for *x*-ray structure determination were recrystallized from CH_2_Cl_2_/CH_3_OH (4:1 v/v) by the slow evaporation of the solvent at room temperature after several days, Mp. 410-411 K (decomposition).

### Refinement

Hydrogen atoms attached to C15 and hydroxyl H atom were located from the difference map and refined isotropically. The remaining H atoms were placed in calculated positions with (C—H) = 0.93 for aromatic and CH, 0.97 for CH_2_ and 0.96 Å for CH_3_ atoms. The *U*_iso_ values were constrained to be 1.5*U*_eq_ of the carrier atom for methyl H atoms and 1.2*U*_eq_ for the remaining H atoms. A rotating group model was used for the methyl groups. The highest residual electron density peak is located at 1.51 Å from H16C and the deepest hole is located at 1.43 Å from C11.

### Crystal data

**Table:**

C_19_H_20_O_5_	*F*(000) = 696
*M**_r_* = 328.35	*D*_x_ = 1.361 Mg m^−^^3^
Monoclinic, *P*2_1_/*c*	Melting point = 410–411 K
Hall symbol: -P 2ybc	Cu *K*α radiation, λ = 1.54178 Å
*a* = 10.2239 (2) Å	Cell parameters from 3114 reflections
*b* = 11.3090 (3) Å	θ = 5.8–72.0°
*c* = 13.8764 (3) Å	µ = 0.81 mm^−^^1^
β = 93.108 (1)°	*T* = 100 K
*V* = 1602.06 (6) Å^3^	Block, yellow
*Z* = 4	0.43 × 0.43 × 0.33 mm

### Data collection

**Table:**

Bruker APEX DUO CCD area-detector diffractometer	3114 independent reflections
Radiation source: sealed tube	3088 reflections with *I* > 2σ(*I*)
graphite	*R*_int_ = 0.027
φ and ω scans	θ_max_ = 72.0°, θ_min_ = 5.8°
Absorption correction: multi-scan (*SADABS*; Bruker, 2009)	*h* = −12→12
*T*_min_ = 0.721, *T*_max_ = 0.774	*k* = −12→13
48432 measured reflections	*l* = −16→16

### Refinement

**Table:**

Refinement on *F*^2^	Secondary atom site location: difference Fourier map
Least-squares matrix: full	Hydrogen site location: inferred from neighbouring sites
*R*[*F*^2^ > 2σ(*F*^2^)] = 0.047	H atoms treated by a mixture of independent and constrained refinement
*wR*(*F*^2^) = 0.147	*w* = 1/[σ^2^(*F*_o_^2^) + (0.085*P*)^2^ + 0.4129*P*] where *P* = (*F*_o_^2^ + 2*F*_c_^2^)/3
*S* = 1.29	(Δ/σ)_max_ = 0.001
3114 reflections	Δρ_max_ = 0.71 e Å^−^^3^
234 parameters	Δρ_min_ = −0.84 e Å^−^^3^
0 restraints	Extinction correction: *SHELXTL* (Sheldrick, 2008), Fc^*^=kFc[1+0.001xFc^2^λ^3^/sin(2θ)]^-1/4^
Primary atom site location: structure-invariant direct methods	Extinction coefficient: 0.041 (2)

### Special details

**Table:**

Experimental. The crystal was placed in the cold stream of an Oxford Cryosystems Cobra open-flow nitrogen cryostat (Cosier & Glazer, 1986) operating at 100.0 (1) K.
Geometry. All esds (except the esd in the dihedral angle between two l.s. planes) are estimated using the full covariance matrix. The cell esds are taken into account individually in the estimation of esds in distances, angles and torsion angles; correlations between esds in cell parameters are only used when they are defined by crystal symmetry. An approximate (isotropic) treatment of cell esds is used for estimating esds involving l.s. planes.
Refinement. Refinement of F^2^ against ALL reflections. The weighted R-factor wR and goodness of fit S are based on F^2^, conventional R-factors R are based on F, with F set to zero for negative F^2^. The threshold expression of F^2^ > 2sigma(F^2^) is used only for calculating R-factors(gt) etc. and is not relevant to the choice of reflections for refinement. R-factors based on F^2^ are statistically about twice as large as those based on F, and R- factors based on ALL data will be even larger.

### Fractional atomic coordinates and isotropic or equivalent isotropic displacement parameters (Å^2^)

**Table:**

	*x*	*y*	*z*	*U*_iso_*/*U*_eq_	
O1	0.24205 (9)	1.16069 (8)	0.99936 (7)	0.0182 (3)	
O2	0.25452 (9)	0.76187 (8)	0.90044 (6)	0.0178 (3)	
O3	0.25906 (11)	1.34713 (9)	1.04469 (8)	0.0251 (3)	
O4	0.01977 (10)	0.66004 (9)	1.11316 (7)	0.0228 (3)	
O5	0.01904 (10)	0.87092 (9)	1.17530 (7)	0.0216 (3)	
H1O5	0.010 (2)	0.789 (2)	1.1695 (16)	0.046 (6)*	
C1	0.20676 (12)	1.04507 (12)	1.01109 (9)	0.0155 (3)	
C2	0.21353 (13)	1.25150 (12)	1.06206 (10)	0.0188 (3)	
C3	0.13341 (13)	1.22145 (13)	1.14119 (10)	0.0201 (3)	
H3A	0.1083	1.2802	1.1833	0.024*	
C4	0.09493 (13)	1.10932 (12)	1.15431 (10)	0.0186 (3)	
H4A	0.0440	1.0912	1.2059	0.022*	
C5	0.13116 (12)	1.01699 (12)	1.09000 (9)	0.0164 (3)	
C6	0.09425 (12)	0.89887 (12)	1.10224 (9)	0.0165 (3)	
C7	0.13559 (12)	0.81207 (12)	1.03860 (9)	0.0162 (3)	
C8	0.09306 (12)	0.69006 (12)	1.04907 (10)	0.0180 (3)	
C9	0.13851 (13)	0.60284 (12)	0.97689 (10)	0.0194 (3)	
H9A	0.0724	0.5956	0.9244	0.023*	
H9B	0.1490	0.5260	1.0074	0.023*	
C10	0.26803 (14)	0.63988 (11)	0.93635 (10)	0.0183 (3)	
C11	0.21402 (12)	0.84528 (12)	0.96178 (9)	0.0154 (3)	
C12	0.25049 (12)	0.96284 (12)	0.94385 (9)	0.0154 (3)	
C13	0.33249 (13)	0.98950 (12)	0.85555 (9)	0.0176 (3)	
C14	0.45881 (13)	0.91792 (13)	0.86498 (10)	0.0211 (3)	
H14A	0.5002	0.9134	0.9262	0.025*	
C15	0.51505 (15)	0.86207 (14)	0.79547 (11)	0.0258 (4)	
H15A	0.473 (2)	0.8581 (18)	0.7301 (16)	0.038 (5)*	
H15B	0.598 (2)	0.8228 (18)	0.8085 (14)	0.034 (5)*	
C16	0.24997 (13)	0.95973 (13)	0.76241 (9)	0.0204 (3)	
H16A	0.2977	0.9812	0.7073	0.031*	
H16B	0.1691	1.0029	0.7614	0.031*	
H16C	0.2318	0.8765	0.7605	0.031*	
C17	0.37739 (16)	1.11907 (13)	0.84519 (11)	0.0273 (4)	
H17A	0.4288	1.1263	0.7896	0.041*	
H17B	0.4293	1.1419	0.9019	0.041*	
H17C	0.3020	1.1695	0.8377	0.041*	
C18	0.29707 (16)	0.56754 (13)	0.84802 (11)	0.0252 (3)	
H18A	0.3768	0.5951	0.8222	0.038*	
H18B	0.2263	0.5759	0.8001	0.038*	
H18C	0.3065	0.4858	0.8658	0.038*	
C19	0.38167 (14)	0.63524 (12)	1.01187 (10)	0.0213 (3)	
H19A	0.4603	0.6615	0.9837	0.032*	
H19B	0.3932	0.5555	1.0345	0.032*	
H19C	0.3631	0.6857	1.0650	0.032*	

### Atomic displacement parameters (Å^2^)

**Table:**

	*U*^11^	*U*^22^	*U*^33^	*U*^12^	*U*^13^	*U*^23^
O1	0.0233 (5)	0.0130 (5)	0.0188 (5)	−0.0003 (4)	0.0045 (4)	−0.0003 (3)
O2	0.0241 (5)	0.0126 (5)	0.0169 (5)	0.0009 (4)	0.0044 (4)	−0.0005 (3)
O3	0.0334 (6)	0.0144 (5)	0.0278 (6)	−0.0006 (4)	0.0039 (4)	−0.0006 (4)
O4	0.0219 (5)	0.0208 (5)	0.0261 (5)	−0.0027 (4)	0.0059 (4)	0.0046 (4)
O5	0.0235 (5)	0.0213 (6)	0.0208 (5)	−0.0009 (4)	0.0088 (4)	0.0018 (4)
C1	0.0153 (6)	0.0140 (6)	0.0171 (6)	0.0002 (5)	−0.0007 (5)	0.0011 (5)
C2	0.0205 (7)	0.0156 (7)	0.0201 (7)	0.0024 (5)	−0.0015 (5)	−0.0020 (5)
C3	0.0209 (7)	0.0203 (7)	0.0191 (7)	0.0042 (5)	0.0011 (5)	−0.0048 (5)
C4	0.0165 (6)	0.0225 (7)	0.0168 (6)	0.0024 (5)	0.0018 (5)	−0.0015 (5)
C5	0.0156 (6)	0.0183 (7)	0.0153 (6)	0.0013 (5)	0.0010 (5)	0.0002 (5)
C6	0.0139 (6)	0.0210 (7)	0.0147 (6)	0.0006 (5)	0.0012 (5)	0.0019 (5)
C7	0.0154 (6)	0.0164 (7)	0.0166 (6)	0.0001 (5)	0.0002 (5)	0.0019 (5)
C8	0.0150 (6)	0.0183 (7)	0.0204 (7)	0.0003 (5)	−0.0015 (5)	0.0033 (5)
C9	0.0200 (7)	0.0138 (6)	0.0242 (7)	−0.0017 (5)	0.0011 (5)	0.0014 (5)
C10	0.0227 (7)	0.0120 (6)	0.0206 (7)	0.0008 (5)	0.0028 (5)	0.0009 (5)
C11	0.0152 (6)	0.0161 (7)	0.0148 (6)	0.0014 (5)	−0.0004 (5)	−0.0009 (5)
C12	0.0159 (6)	0.0157 (7)	0.0147 (6)	0.0003 (5)	0.0013 (5)	0.0007 (5)
C13	0.0203 (7)	0.0170 (7)	0.0160 (6)	0.0000 (5)	0.0052 (5)	−0.0003 (5)
C14	0.0180 (7)	0.0267 (7)	0.0187 (7)	−0.0006 (5)	0.0011 (5)	0.0007 (5)
C15	0.0217 (7)	0.0305 (8)	0.0250 (8)	0.0060 (6)	0.0003 (6)	−0.0025 (6)
C16	0.0217 (7)	0.0233 (7)	0.0165 (7)	0.0032 (5)	0.0030 (5)	0.0028 (5)
C17	0.0376 (9)	0.0201 (7)	0.0257 (7)	−0.0052 (6)	0.0160 (6)	−0.0010 (6)
C18	0.0338 (8)	0.0174 (7)	0.0248 (7)	0.0017 (6)	0.0048 (6)	−0.0037 (5)
C19	0.0204 (7)	0.0199 (7)	0.0237 (7)	0.0020 (5)	0.0028 (5)	0.0017 (5)

### Geometric parameters (Å, °)

**Table:**

O1—C1	1.3685 (16)	C10—C18	1.5164 (19)
O1—C2	1.3872 (16)	C10—C19	1.5227 (19)
O2—C11	1.3505 (16)	C11—C12	1.4065 (19)
O2—C10	1.4708 (15)	C12—C13	1.5513 (17)
O3—C2	1.2069 (18)	C13—C14	1.5238 (19)
O4—C8	1.2410 (17)	C13—C16	1.5419 (18)
O5—C6	1.3430 (16)	C13—C17	1.5445 (18)
O5—H1O5	0.93 (2)	C14—C15	1.311 (2)
C1—C12	1.4073 (19)	C14—H14A	0.9300
C1—C5	1.4103 (18)	C15—H15A	0.98 (2)
C2—C3	1.4456 (19)	C15—H15B	0.97 (2)
C3—C4	1.343 (2)	C16—H16A	0.9600
C3—H3A	0.9300	C16—H16B	0.9600
C4—C5	1.4352 (18)	C16—H16C	0.9600
C4—H4A	0.9300	C17—H17A	0.9600
C5—C6	1.4009 (19)	C17—H17B	0.9600
C6—C7	1.4009 (19)	C17—H17C	0.9600
C7—C11	1.4189 (18)	C18—H18A	0.9600
C7—C8	1.4564 (18)	C18—H18B	0.9600
C8—C9	1.4975 (19)	C18—H18C	0.9600
C9—C10	1.5253 (19)	C19—H19A	0.9600
C9—H9A	0.9700	C19—H19B	0.9600
C9—H9B	0.9700	C19—H19C	0.9600
			
C1—O1—C2	124.55 (11)	C12—C11—C7	123.30 (12)
C11—O2—C10	117.90 (10)	C11—C12—C1	114.24 (12)
C6—O5—H1O5	103.0 (14)	C11—C12—C13	118.90 (11)
O1—C1—C12	117.21 (12)	C1—C12—C13	126.86 (12)
O1—C1—C5	117.81 (12)	C14—C13—C16	112.25 (11)
C12—C1—C5	124.97 (13)	C14—C13—C17	104.91 (11)
O3—C2—O1	116.21 (12)	C16—C13—C17	106.32 (11)
O3—C2—C3	127.09 (13)	C14—C13—C12	108.70 (11)
O1—C2—C3	116.70 (12)	C16—C13—C12	108.97 (11)
C4—C3—C2	120.48 (12)	C17—C13—C12	115.72 (11)
C4—C3—H3A	119.8	C15—C14—C13	126.59 (13)
C2—C3—H3A	119.8	C15—C14—H14A	116.7
C3—C4—C5	121.02 (13)	C13—C14—H14A	116.7
C3—C4—H4A	119.5	C14—C15—H15A	120.7 (12)
C5—C4—H4A	119.5	C14—C15—H15B	120.0 (12)
C6—C5—C1	118.12 (12)	H15A—C15—H15B	119.3 (17)
C6—C5—C4	122.55 (12)	C13—C16—H16A	109.5
C1—C5—C4	119.32 (12)	C13—C16—H16B	109.5
O5—C6—C7	121.03 (12)	H16A—C16—H16B	109.5
O5—C6—C5	119.01 (12)	C13—C16—H16C	109.5
C7—C6—C5	119.95 (12)	H16A—C16—H16C	109.5
C6—C7—C11	119.36 (12)	H16B—C16—H16C	109.5
C6—C7—C8	119.95 (12)	C13—C17—H17A	109.5
C11—C7—C8	120.64 (12)	C13—C17—H17B	109.5
O4—C8—C7	121.72 (13)	H17A—C17—H17B	109.5
O4—C8—C9	121.36 (12)	C13—C17—H17C	109.5
C7—C8—C9	116.89 (12)	H17A—C17—H17C	109.5
C8—C9—C10	111.89 (11)	H17B—C17—H17C	109.5
C8—C9—H9A	109.2	C10—C18—H18A	109.5
C10—C9—H9A	109.2	C10—C18—H18B	109.5
C8—C9—H9B	109.2	H18A—C18—H18B	109.5
C10—C9—H9B	109.2	C10—C18—H18C	109.5
H9A—C9—H9B	107.9	H18A—C18—H18C	109.5
O2—C10—C18	104.54 (11)	H18B—C18—H18C	109.5
O2—C10—C19	108.64 (11)	C10—C19—H19A	109.5
C18—C10—C19	111.23 (12)	C10—C19—H19B	109.5
O2—C10—C9	108.37 (11)	H19A—C19—H19B	109.5
C18—C10—C9	111.21 (12)	C10—C19—H19C	109.5
C19—C10—C9	112.46 (11)	H19A—C19—H19C	109.5
O2—C11—C12	117.10 (12)	H19B—C19—H19C	109.5
O2—C11—C7	119.58 (12)		
			
C2—O1—C1—C12	−176.20 (11)	C11—O2—C10—C19	−68.22 (14)
C2—O1—C1—C5	2.95 (18)	C11—O2—C10—C9	54.25 (14)
C1—O1—C2—O3	175.33 (12)	C8—C9—C10—O2	−52.54 (14)
C1—O1—C2—C3	−4.33 (18)	C8—C9—C10—C18	−166.91 (11)
O3—C2—C3—C4	−176.61 (14)	C8—C9—C10—C19	67.58 (14)
O1—C2—C3—C4	3.01 (19)	C10—O2—C11—C12	154.89 (11)
C2—C3—C4—C5	−0.5 (2)	C10—O2—C11—C7	−26.57 (16)
O1—C1—C5—C6	179.76 (11)	C6—C7—C11—O2	179.76 (11)
C12—C1—C5—C6	−1.2 (2)	C8—C7—C11—O2	−3.05 (18)
O1—C1—C5—C4	−0.19 (18)	C6—C7—C11—C12	−1.79 (19)
C12—C1—C5—C4	178.89 (12)	C8—C7—C11—C12	175.40 (11)
C3—C4—C5—C6	179.13 (12)	O2—C11—C12—C1	−179.17 (10)
C3—C4—C5—C1	−0.9 (2)	C7—C11—C12—C1	2.35 (19)
C1—C5—C6—O5	−178.07 (11)	O2—C11—C12—C13	0.61 (17)
C4—C5—C6—O5	1.87 (19)	C7—C11—C12—C13	−177.87 (11)
C1—C5—C6—C7	1.80 (19)	O1—C1—C12—C11	178.22 (10)
C4—C5—C6—C7	−178.26 (11)	C5—C1—C12—C11	−0.87 (19)
O5—C6—C7—C11	179.45 (11)	O1—C1—C12—C13	−1.54 (19)
C5—C6—C7—C11	−0.42 (19)	C5—C1—C12—C13	179.37 (12)
O5—C6—C7—C8	2.24 (19)	C11—C12—C13—C14	−57.89 (15)
C5—C6—C7—C8	−177.63 (11)	C1—C12—C13—C14	121.86 (14)
C6—C7—C8—O4	0.37 (19)	C11—C12—C13—C16	64.73 (15)
C11—C7—C8—O4	−176.81 (12)	C1—C12—C13—C16	−115.52 (14)
C6—C7—C8—C9	178.52 (11)	C11—C12—C13—C17	−175.57 (12)
C11—C7—C8—C9	1.35 (18)	C1—C12—C13—C17	4.2 (2)
O4—C8—C9—C10	−154.56 (12)	C16—C13—C14—C15	18.3 (2)
C7—C8—C9—C10	27.28 (16)	C17—C13—C14—C15	−96.71 (17)
C11—O2—C10—C18	172.93 (11)	C12—C13—C14—C15	138.93 (16)

### Hydrogen-bond geometry (Å, °)

**Table:**

Cg1 is the centroid of the C1–C5/O1ring.

**Table:**

*D*—H···*A*	*D*—H	H···*A*	*D*···*A*	*D*—H···*A*
O5—H1O5···O4	0.93 (2)	1.66 (2)	2.5361 (14)	155 (2)
C9—H9B···O3^i^	0.97	2.36	3.2621 (17)	155
C16—H16B···O5^ii^	0.96	2.59	3.4982 (17)	159
C16—H16C···O2	0.96	2.34	2.9441 (16)	121
C15—H15B···Cg1^iii^	0.97 (2)	2.83 (2)	3.5908 (16)	136.7 (15)

Symmetry codes:  (i) *x*, *y*−1, *z*; (ii) −*x*, −*y*+2, −*z*+2; (iii) −*x*+1, −*y*+2, −*z*+2.

## References

[bb1] Allen, F. H., Kennard, O., Watson, D. G., Brammer, L., Orpen, A. G. & Taylor, R. (1987). *J. Chem. Soc. Perkin Trans. 2*, pp. S1–19.

[bb2] Bernstein, J., Davis, R. E., Shimoni, L. & Chang, N.-L. (1995). *Angew. Chem. Int. Ed. Engl* **34**, 1555–1573.

[bb3] Bruker (2009). *APEX2*, *SAINT* and *SADABS* Bruker AXS Inc., Madison, Wisconsin, USA.

[bb4] Cremer, D. & Pople, J. A. (1975). *J. Am. Chem. Soc.* **97**, 1354–1358.

[bb5] Huang, S.-C., Wu, P.-L. & Wu, T.-S. (1997). *Phytochemistry*, **44**, 179–181.

[bb6] Kongkathip, B., Kongkathip, N., Sunthitikawinsakul, A., Napaswat, C. & Yoosook, C. (2005). *Phytother. Res.* **19**, 728–731. 10.1002/ptr.1738 16177980

[bb7] Laphookhieo, S., Sripisut, T., Prawat, U. & Karalai, C. (2009). *Heterocycles*, **78**, 2115–2119.

[bb8] Maneerat, W., Prawat, U., Saewan, N. & Laphookhieo, S. (2010). *J. Braz. Chem. Soc* **21**, 665–668.

[bb9] Sheldrick, G. M. (2008). *Acta Cryst.* A**64**, 112–122. 10.1107/S0108767307043930 18156677

[bb10] Spek, A. L. (2009). *Acta Cryst.* D**65**, 148–155. 10.1107/S090744490804362X PMC2631630 19171970

[bb11] Su, C.-R., Yeh, S.-F., Liu, C.-M., Damu, A.-G., Kuo, T.-H., Chaing, P.-C., Bastow, K. F., Lee, K.-H. & Wu, T.-S. (2009). *Bioorg. Med. Chem* **17**, 6137–6143. 10.1016/j.bmc.2008.12.007 19635670

[bb12] Tangyuenyongwatthana, P., Pummangura, S. & Thanyavuthi, D. (1992). *Songklanakarin J. Sci. Technol* **14**, 157–162.

[bb13] Yenjai, C., Sripontan, S., Sriprajun, P., Kittakoop, P., Jintasirikul, A., Tanticharoen, M. & Thebtaranonth, Y. (2000). *Planta Med* **66**, 277–279. 10.1055/s-2000-8558 10821058

